# Diffusion in Porous Rock Is Anomalous

**DOI:** 10.1021/acs.est.4c01386

**Published:** 2024-05-13

**Authors:** Ashish Rajyaguru, Ralf Metzler, Ishai Dror, Daniel Grolimund, Brian Berkowitz

**Affiliations:** † Department of Earth and Planetary Sciences, Weizmann Institute of Science, Rehovot 7610001, Israel; ‡ Institute for Physics and Astronomy, University of Potsdam, 14476 Potsdam, Germany; § Asia Pacific Centre for Theoretical Physics, Pohang 37673, Republic of Korea; ∥ Paul Scherrer Institut, 5232 Villigen, Switzerland

**Keywords:** non-Fickian diffusion, chemical diffusion, breakthrough curve, power law

## Abstract

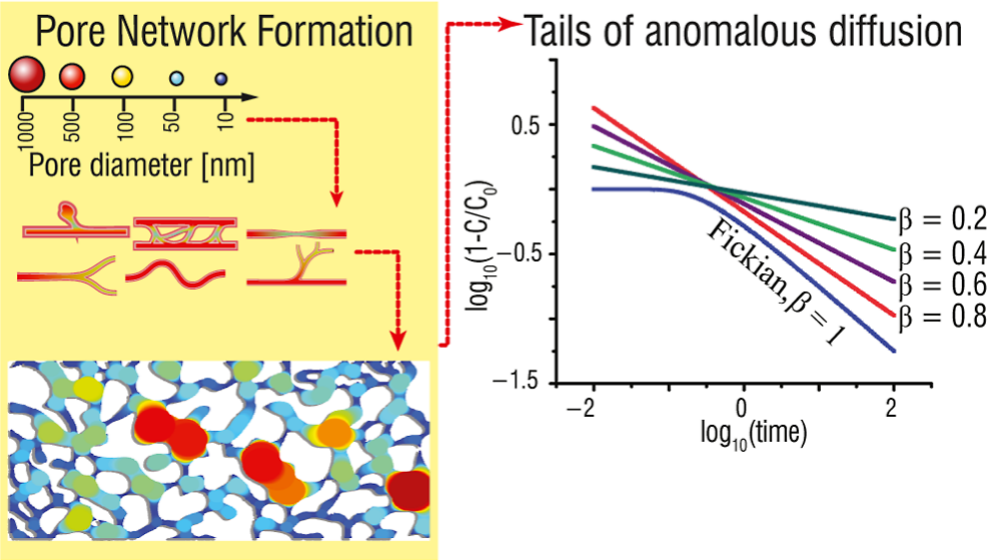

Molecular diffusion of chemical species in subsurface environments—rock formations, soil sediments, marine, river, and lake sediments—plays a critical role in a variety of dynamic processes, many of which affect water chemistry. We investigate and demonstrate the occurrence of anomalous (non-Fickian) diffusion behavior, distinct from classically assumed Fickian diffusion. We measured molecular diffusion through a series of five chalk and dolomite rock samples over a period of about two months. We demonstrate that in all cases, diffusion behavior is significantly different than Fickian. We then analyze the results using a continuous time random walk framework that can describe anomalous diffusion in heterogeneous porous materials such as rock. This methodology shows extreme long-time tailing of tracer advance as compared to conventional Fickian diffusion processes. The finding that distinct anomalous diffusion occurs ubiquitously implies that diffusion-driven processes in subsurface zones should be analyzed using tools that account for non-Fickian diffusion.

## Abstract 2

Chemical diffusion in porous rock is usually considered a Fickian process. We measure and model the occurrence of pronounced non-Fickian diffusion, with extreme long-time chemical tailing, implying the need to (re)analyze diffusion accordingly.

## Introduction

Diffusion is fundamental in a wide range of phenomena across disciplines. Whether in biological processes, chemical reactions, material science, or environmental dynamics, diffusion is a basic mechanism that governs system behavior at different scales. Accurate quantification of molecular diffusion—diffusion-controlled transport—in water-saturated geological formations, and in marine, river, and lake sediments, is key to assessing rates and timing of chemical arrivals at critical locations of interest, as well as to interpreting patterns of chemical reaction and precipitation. Similarly, assessing molecular diffusion in subsurface geological formations is critical in the context of developing subsurface disposal sites for radioactive waste or anthropogenic CO_2_ storage and in mine waste recovery. In many cases, diffusion-controlled transport occurs in porous media that range from essentially impermeable rock-like claystone to loosely compacted sands, each of which exhibits a unique pore structure.

### How Is Diffusion Usually Quantified?

The temporal spreading of Brownian particles in a free fluid (liquid or gas) is described by a Gaussian law for the probability density function. While flickering of coal dust particles on an alcohol surface was noted by Ingenhousz in 1785 and irregular movement of small pollen grains was observed under a microscope by Brown in 1827,^1^ Fick^2^ provided the first quantification of this process in 1855.

Fick’s second law, embodied in the classical diffusion equation, states that in a macroscopically one-dimensional (1D) domain
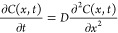
1where *C* is concentration, *x* and *t* are distance and time, respectively, and *D* represents a coefficient of molecular diffusion. In solving this equation, the spreading pattern of diffusing particles or chemical species is characterized by a mean squared displacement that scales linearly with time: ⟨*x*^2^(*t*)⟩ = 2*Dt*, for initial particle positions centered at *x*_0_ = 0.^3,4^ This expression was first verified experimentally by Nordlund,^5^ and then subsequently by others, for molecular diffusion *in a free fluid*.

But how do particles diffuse in “crowded environments” such as, e.g., water-saturated porous rock, biological tissues and cells, and dense liquids and gels? Does diffusion differ from Gaussian behavior, and if so, how can we quantify it?

Indeed, deviations from Fickian diffusion (and Brownian motion) are widespread across disciplines. Examples of anomalous diffusion arise during passive tracer particle movement^6−8^ and molecular motor-driven and nanoparticle motion in biological cells,^9−12^ particle motion in crowded environments such as biological membranes^13−15^ or dense liquids,^16,17^ nanoparticle diffusion in porous media,^18,19^ and transport in gels.^11,20−22^ In these cases, the spreading pattern of diffusing particles is characterized by a mean squared displacement, ⟨*x*^2^(*t*)⟩, that does not scale linearly with time. And yet, in naturally occurring geological materials, molecular diffusion of chemical species, in the absence of an advective flow field, is almost invariably modeled as a Brownian process, quantified with eq 1. Somewhat surprisingly, the possible—or likely—occurrence of anomalous diffusion in such heterogeneous, disordered media has been almost completely ignored.

### Existing Measurements of Diffusion in Geological Materials

In Earth science fields that involve the study of chemical diffusion processes in water-saturated geological materials, Fick’s law (eq 1) is almost always assumed to represent diffusive processes, with the diffusion coefficient (dimensions of length^2^/time) in a free liquid, *D*, being replaced by an “effective porous medium diffusion coefficient”, *D*_d_, that is assumed to account for the effects of tortuosity, τ, and porosity, *n* of the porous medium under consideration; i.e., one generally assumes *D*_d_ = *D*·*f*(τ, *n*), where *f*(τ, *n*) is an empirical function. The literature contains a wide variety of analyses to “define” *f*(τ, *n*) explicitly or to specify *D*_d_ relative to *D* of a chemical species as determined for a free liquid, but the results largely remain empirical.

With these assumptions, a limited number of experimental measurements to estimate the actual values of *D*_d_ have been reported. These experiments generally involve the use of a diffusion cell in which a section of (water-saturated) porous rock, often a cylindrical core of diameter 25–75 mm and thickness 7–25 mm is sandwiched between inlet and outlet reservoirs of liquid;^23−32^ at the start of an experiment, the inlet reservoir liquid contains an inert chemical tracer (e.g., bromide) with a specified concentration *C*_0_, while the initial concentration in the outlet reservoir and in the section of fully liquid saturated porous rock is *C* = 0.

The inlet and outlet tracer concentrations are then monitored over time, to yield a breakthrough curve (BTC) of concentration versus time. Solutions of eq 1 are then fitted to the BTC to estimate a value of *D*_d_. Analytical solutions of Fick’s law are “most convenient” when considering diffusion along a semi-infinite domain, wherein the outlet plane is sufficiently far from the inlet reservoir such that *C* = 0 even at very long times. However, the small dimensions of the experimental setups described above enable the outlet concentration to increase significantly, e.g., up to *C*/*C*_0_ = 0.65, while the inlet reservoir concentration decreases accordingly, over experiment durations of ∼30–100 days.^23,24,28,32^ Fitting the BTCs measured in such situations requires the use of (nonsemi-infinite boundary condition) solutions that account for the evolving equilibration between inlet and outlet.^33^

There are two principal drawbacks associated with this experimental approach. First, the “force-fit” of eq 1 solutions to measurements presupposes the diffusion to be Fickian, and does not allow for the possible occurrence of anomalous diffusion. Second, these experiments “blur” the long-time tailing because the evolving concentration at the outlet boundary is equilibrating with the inlet concentration. Experimental setups to discern possible anomalous diffusion behavior require measurements that clearly and definitively assess the slope of the long-time tailing of the BTCs.

Aside from experiments, other studies that quantify molecular diffusion behavior either focus on variations of (Fickian) diffusion formulations (based on eq 1),^34−37^ or involve numerical simulations of Fickian diffusion with time-dependent and spatially variable diffusion coefficients.^38,39^

Here, we use specially designed diffusion cells to measure molecular diffusion through a set of five chalk and dolomite rock samples, over a period of about 2 months. The experimental setup was designed specifically to mimic environmentally realistic scenarios with an essentially semi-infinite boundary condition. The results demonstrate, in all cases, diffusion behavior that is distinctly not Fickian, unlike diffusion that occurs in a free liquid. We then interpret the results using a recently developed theoretical analysis, based on a continuous time random walk (CTRW) framework tailored specifically for such a scenario, that can effectively quantify anomalous *diffusion* in porous materials such as rock. Diffusion that follows such anomalous behavior shows extended long-time tailing of tracer advance as compared to conventional Fickian diffusion processes. We discuss the implications of these findings in terms of how anomalous diffusion can impact diffusion-driven processes in subsurface zones.

## Methods and Materials

### Diffusion Cell Setup

**Figure 1:**
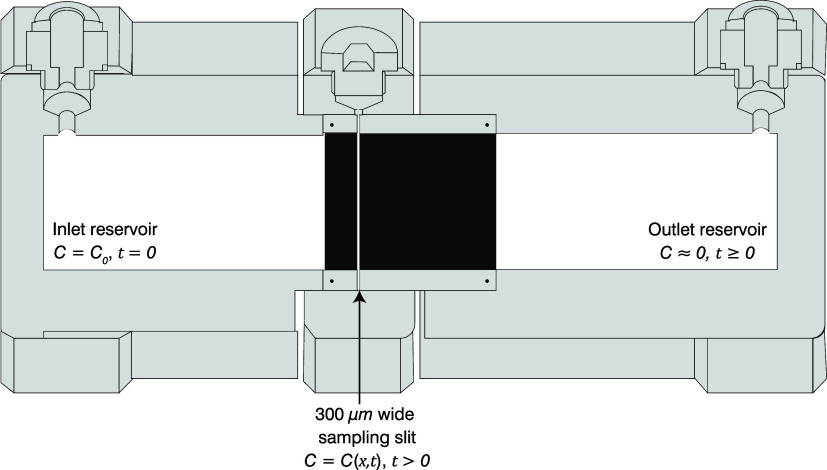
Schematic outline of the diffusion cell.

A schematic representation of the diffusion cells used in this study is illustrated in Figure 1. Each cell was composed of two 50 mL reservoirs, sandwiching a 35 mm cylindrical diameter rock core cut into two sections, of lengths 10 and 35 mm. The 10 mm rock core was placed in the bore of the inlet reservoir, and the 35 mm rock core was placed in the bore of the outlet reservoir. A central rim was then placed between the open surfaces of the two rock cores, and the cell was closed by 8 screws. The geometry of the central rim was fabricated such that after closing the diffusion cell, a thin slit of 300 μm remained between the two rock samples; the slit size was sufficient to insert a needle to extract water samples to measure tracer concentration.

### Experimental Protocols

Following diffusion cell assembly, each cell was placed vertically, with the inlet reservoir on the lower end, into a large container of “pore water”. The water level in the container was raised slowly, over a period of 1 month, to allow water to slowly saturate the rock samples and fill the outlet reservoir; this protocol, as reported previously,^32^ simulates the water level rise in bedrock in natural environments. To ensure full saturation and equilibration between rock pore surfaces and pore fluid, the cells were allowed to equilibrate for an additional 1 month, after which the inlet and outlet reservoir screws were inserted to seal the diffusion cells; the cells were then removed from the large reservoir. At this stage, 500 μL of pore water solution was injected in the central slit of each cell, and its stability was monitored for an additional 15 days. This simple test ensured that the samples were fully saturated and with no leaks (no change in slit volume).

The synthetic pore water chemistry was chosen to mimic the ion concentrations commonly observed in carbonate rocks.^28,32^ The ionic composition was: NaCl (159.01 mM), MgCl_2_·6H_2_O (0.07 mM), NaHCO_3_ (0.39 mM), CaCl_2_·2H_2_O (20.62 mM), and pCO_2_ (−3.5 atm). The constant-background electrolyte concentration reduces changes in pH and the possible codiffusion of other ionic species.

To begin the diffusion experiments, the inlet reservoir solution was replaced with a fresh pore water solution that also contained 100 mM NaBr; the slit and outlet reservoir solutions were also replaced with a fresh pore water solution. Throughout the experiments, the diffusion cells were placed vertically with the inlet reservoir containing the bromide pore water solution at the bottom. This approach ensured that bromide diffused naturally, upward through the rock samples, and minimized any possible impacts of gravity and density distribution. Over the course of about 2 months, each experiment involved periodic sampling of 20 μL of slit solution and replacement of the same volume with a bromide free solution. The process of diffusion generally exhibits an initial transient evolution followed by longer time concentration tailing behavior. In the current experiments, the initial phase extended over ∼20 days. The sampling protocol was as follows: sampling every 2–4 h in the first week, every 6 h during the second week, every 8 h during the third week, every 12 h during the fourth week, and every 24 h thereafter until experiment completion (60–67 days).

To further explain the diffusion cell design, it should be recognized that in natural systems, a typical scenario of aquifer or biosphere contamination is the (usually) accidental release of chemical species from a source, which can be in the form of a 1D, 2D, or 3D source (e.g., a well, a surface landfill, or a subsurface repository, respectively). A chemical species is thus considered a contaminant, i.e., a species not generally native to the particular geological setting of interest (e.g., a subsurface geological formation or a marine, river, or lake sediment). In such cases, a chemical species diffusing from a source will generally continue to advance via the natural concentration gradient, without a concurrent concentration increase in a “downstream reservoir”, but rather subject to an essentially semi-infinite *C*(∞,*t*) = 0 boundary condition. Similar scenarios apply to geochemical investigation of “natural” chemical species, such as iron, lead, or strontium, diffusing through a geological layer or sediment, and without or with chemical reactions such as adsorption or precipitation. The diffusion cells were therefore designed and built to simulate tracer diffusion under semi-infinite boundary conditions, namely, with a constant concentration inlet boundary [*C*(0,*t*) = *C*_0_] and a zero concentration at the outlet boundary [*C*(∞,*t*) = 0], and with the slit acting as the sampling port. Note that the 35 mm rock core between the sampling slit and the outlet reservoir acts as a continuation of the rock, mimicking a single long (45 mm) sample, so that the diffusion cell setup essentially represents a pseudo zero-concentration (semi-infinite) boundary condition.

As the rock samples analyzed here were chosen because of their relatively low permeability, tracer concentration increases in the outlet reservoir were expected to occur over several months or more. Moreover, the small slit volume was chosen to reduce possible dilution effects, to enhance detection of small, long-time changes in concentration.

### Concentration Measurements

The samples extracted from the diffusion cells were analyzed by inductively coupled plasma mass spectrometry (ICP-MS; Agilent 7700s) for bromide concentration. Drift corrections were carried out by analyzing calibration solutions. The standards for the calibration curve included 10, 5, 2, 1, and 0 μM (1029, 514.5, 205.8, 102.9, and 0 ppb NaBr, respectively). During the measurements, ∼20 μL of solution was extracted from the slit and diluted in 4.8 g of double deionized water (DDW, 18.2 MΩ), so that the solution was diluted by a factor of ∼240. For ICP-MS measurements, a second dilution factor of 50 was used. Thus, the sampled solution concentrations were diluted by a factor of 12,000, which reduced the concentration range to that of the calibration curve. Mass-Hunter 4.1 software, version C.01.01, 2015 was used to process ICP-MS data. Post concentration measurements, the data points with RSD > 5% were considered outliers and discarded.

To confirm that the diffusion cell setup yielded essentially constant inlet concentration and zero concentration outlet (semi-infinite) boundary conditions, samples of liquid from the inlet and outlet reservoirs were measured after 2 months at the conclusion of the experiments. The measured inlet and outlet relative concentration values, *C*/*C*_0_(inlet) and *C*/*C*_0_(outlet), respectively, were: desert pink, perpendicular to the bedding plane, 0.95 and 0.04; Edwards Yellow, perpendicular to the bedding plane, 0.96 and 0.07; desert pink, parallel to the bedding plane, 0.96 and 0.03; Edwards Yellow, parallel to the bedding plane, 0.96 and 0.10; and Silurian dolomite, parallel to the bedding plane, 0.98 and 0.02.

### Anomalous Diffusion and the CTRW Modeling Framework

Molecular diffusion in crowded environments has been studied in many contexts,^40^ and the occurrence of “anomalous diffusion”—non-Fickian (or non-Gaussian) diffusion—has been characterized extensively in terms of its long-time scaling behavior.^7−22,41^ In these contexts, it is recognized that diffusion frequently exhibits a power-law dependence ⟨*x*^2^(*t*)⟩ ≃ *D*_β_*t*^β^ on time, characterized by the anomalous diffusion exponent β (with 0 < β < 1), and the generalized diffusion coefficient *D*_β_ (with dimensions length^2^/time^β^). The fractional dimension of time here occurs due to rescaling by a microscopic scaling factor.^42^ Anomalous diffusion, in particular, is frequently revealed in single particle tracking experiments and analyzed in terms of machine-learning approaches.^43,44^

Here, measurements from diffusion cells containing rock samples are analyzed using a CTRW framework developed to describe anomalous diffusion in porous or other “disordered” media. The CTRW framework is applied to anomalous *diffusion*, i.e., systems for which there is no advective flow, only pure diffusion, in porous media. For an effectively 1D, semi-infinite system with an inlet reservoir at a constant chemical concentration, the temporal evolution of the concentration profile in the domain can be quantified. Technically, starting from a CTRW equation with a scale-free waiting time density, the formulation can be recast to a time-fractional diffusion equation. Using this formulation and the connection between the known Brownian solution and its anomalous-diffusive counterpart in terms of the subordination relation, the concentration profiles can be quantified. More specifically, we examine BTCs of concentration versus time, *C*(*x*,*t*) for an effectively 1D, semi-infinite disordered system connected to a reservoir of tracer particles kept at a constant concentration. As a first assessment of the nature of diffusion—Fickian or anomalous—we focus here on asymptotic long-time behavior. Details of the mathematical formulation and derivation of asymptotic solutions are given in Metzler et al.^42^ Solutions for the description of the full evolution of temporal BTCs, at fixed distances from the inlet, remain to be fully determined; they will follow in a future study.

The asymptotic (long-time) scaling behavior, in dimensional form, for a macroscopically 1D system, for Fickian diffusion with β = 1
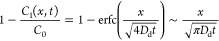
2where erfc is the complementary error function. In contrast, for anomalous diffusion, the asymptotic (long-time) scaling behavior in dimensional form is based on^42^
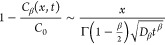
3with Γ being the Gamma function. Thus, under anomalous diffusion, the residual BTC scales as 1 – *C*_β_(*x*,*t*)/*C*_0_ ∼ *t*^–β/2^ as opposed to a ∼*t*^–1/2^ dependence for Fickian diffusion. Log–log plots of 1 – *C*_β_(*x*,*t*)/*C*_0_ versus time *t* yield asymptotes with slopes corresponding to −1/2 and −β/2, respectively, for Fickian (β = 1) and non-Fickian (anomalous, 0 < β < 1) diffusion behavior. With the dimensional expressions eqs 2 and 3, the values of β, *D*_β_, and *D*_d_ can be determined by fitting them to experimental measurements.

## Results and Discussion

### Experimental Measurements and Their Interpretation

A series of five carbonate cores were selected to investigate molecular diffusion under fully water-saturated conditions. All of these rock samples are composed of CaCO_3_ mineral but with different diagenetic histories and ranges of permeability and porosity (Table 1). The cores were further differentiated by sectioning them either parallel or perpendicular to the bedding plane, recognizing that the change in grain matrix orientation represents “secondary” heterogeneity in rock pore structure. The existing measurements and complementary numerical simulations for a wide range of porous media demonstrate the ubiquity of anomalous diffusion, even in relatively “simple” domains;^7−22,41^ it is therefore reasonable to consider the measurements presented here to be representative of diffusion behavior in a broad range of geological settings.

**Table 1:** Rock Type, Properties, and Diffusion Parameters

rock type	porosity^a^ (%)	permeability^a^ (×10^–10^ cm^2^)	β	*D*_β_ (cm^2^/d^β^)	*D*_d_ (cm^2^/d)
desert pink—perpendicular	25–27	2.0–19.7	0.31	0.68	0.05
Edwards yellow—perpendicular	33–35	6.4–8.4	0.40	0.65	0.15
desert pink—parallel	25–27	2.0–19.7	0.26	0.65	0.04
Edwards yellow—parallel	33–35	6.4–8.4	0.49	0.60	0.09
Silurian dolomite—parallel	16–17	3.5–9.9	0.08	1.5	0.17

a Estimated values by supplier (Kocurek Industries Inc., Texas, USA).

The experimental protocol and diffusion cell design (see Section Methods and Materials) enable measurement of the concentration profile *C*(x*,*t*), with x* being a fixed monitoring distance from the inlet; the design mimics an effectively (macroscopically) 1D, semi-infinite system—*C*(∞,*t*) = 0 at the outlet—connected to an inlet reservoir of tracer (bromide) kept at a constant concentration [*C*(0,*t*) = *C*_0_]. These measurements thus determine the tracer BTC at a given distance from the tracer source; here, x* = 10 mm and the outlet is *x* = 45 mm from the inlet. Over the time scale of the experiment, the outlet concentration remained negligible, confirming that the diffusion cell setup represented an essentially semi-infinite (zero concentration) outlet boundary. Full details of the diffusion cell setup and measurement protocol are given in the Section Methods and Materials.

**Figure 2:**
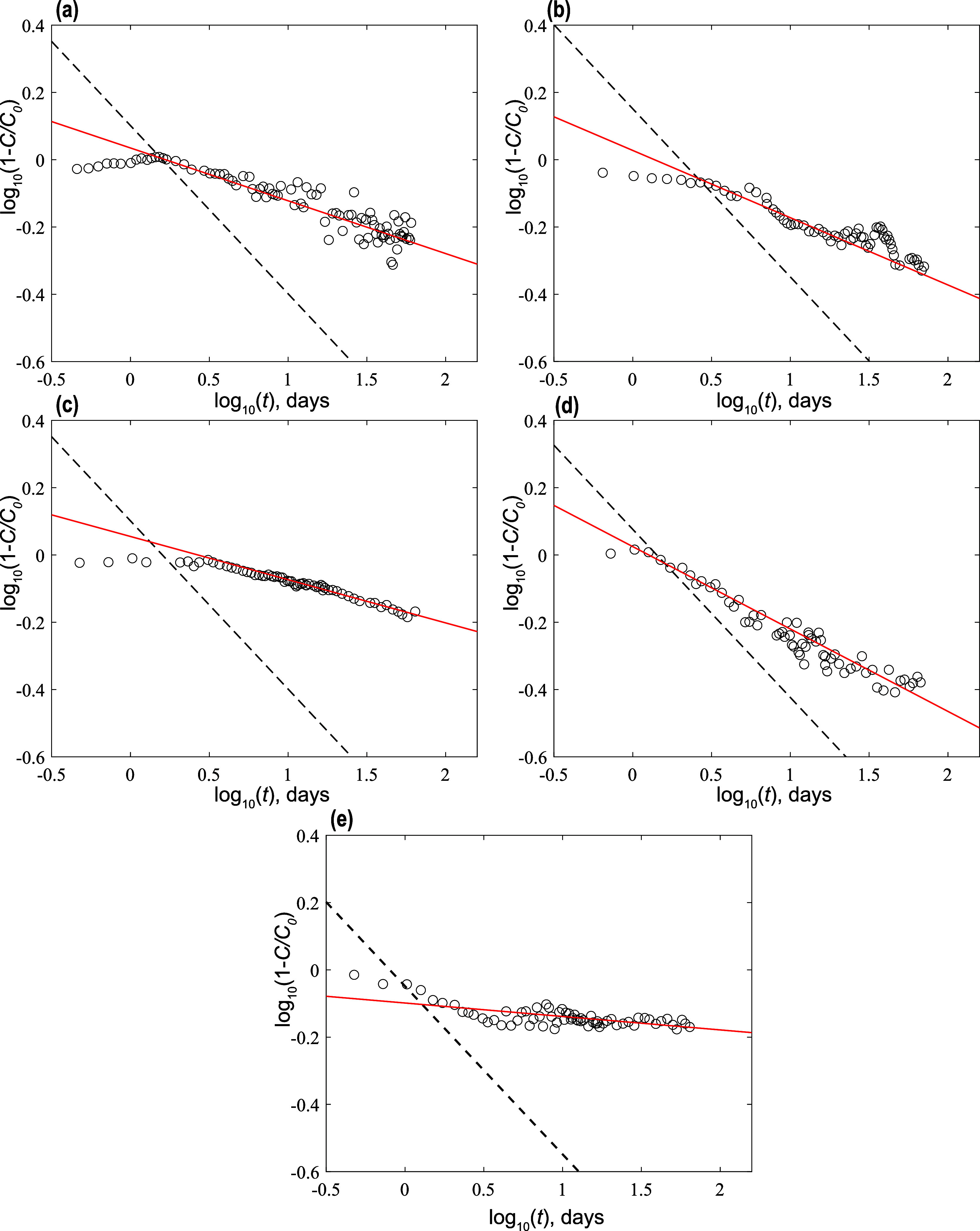
BTC measurements (circles) for tracer diffusion. Data are shown together with a (linear regression) best fit of eq 3 over the asymptotic regime (solid red line), together with a reference Fickian slope of −0.5 (dashed black line). (a) Desert pink, perpendicular to the bedding plane (−β/2 ≈ −0.155 ± 0.012), (b) Edwards yellow, perpendicular to the bedding plane (−β/2 ≈ −0.200 ± 0.012), (c) desert pink, parallel to the bedding plane (−β/2 ≈ −0.130 ± 0.006), (d) Edwards yellow, parallel to the bedding plane (−β/2 ≈ −0.245 ± 0.015), (e) Silurian dolomite, parallel to the bedding plane (−β/2 ≈ −0.04 ± 0.01), where the ± values represent the 95% confidence intervals. *R*^2^ (coefficient of determination) values for the fits are 0.90, 0.78, 0.97, 0.91, and 0.95, respectively for (a–e).

The BTC measurements are plotted on a double logarithmic scale in Figure 2. The data are shown together with a fit of eq 3, together with a reference line showing a Fickian slope of −0.5. Corresponding estimates of β and *D*_β_ from the fitting of eq 3 are shown in Table 1. The fitting procedure was done in two steps—first, a slope of the asymptotic (approximately linear) section of each plot was estimated by linear regression, to fix a value of β, and then eq 3 was employed to estimate *D*_β_ by again fitting (approximated visually) to the data. In this analysis, the values of β and *D*_β_ control, respectively, the slope and the vertical (*y*-axis) position of the asymptote.

The experiments clearly demonstrate that the diffusion is anomalous in all five columns. The long-time (asymptotic) slopes of the “residual” BTCs vary from −β/2 = −0.04 to −0.25, in sharp contrast to the Fickian diffusion slope of −0.5.

It is worth noting, too, from the inspection of the diffusion parameter values shown in Table 1, that while the number of samples is limited and the estimated permeability ranges are broad, there is no clear “correlation” or monotonic trend among values of β and the estimated permeability of each rock, nor between values of β and the direction of (parallel and orthogonal) bedding. On the other hand, higher estimated porosity suggests higher values of β, as might be expected. The highly compacted Silurian dolomite core showed a particularly small β value.

**Figure 3:**
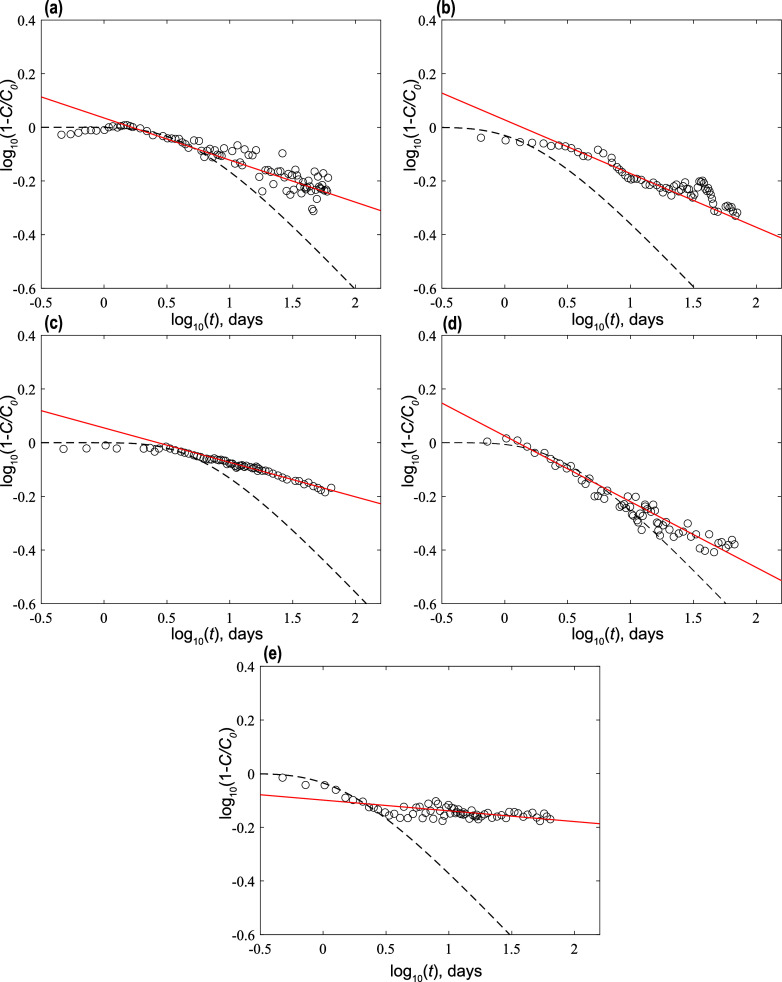
BTC measurements for tracer diffusion. Data are shown together with a best fit of eq 3 (solid red line, as shown also in Figure 2), together with illustrative, full BTC solutions for the dimensional Fickian diffusion case (dashed black line), given by the middle expression of eq 2. Values of *D*_d_ employed here are typical of those estimates for carbonates;^28,32^ see text for discussion. (a) Desert pink, perpendicular to the bedding plane, (b) Edwards yellow, perpendicular to the bedding plane, (c) desert pink, parallel to the bedding plane, (d) Edwards yellow, parallel to the bedding plane, (e) Silurian dolomite, parallel to the bedding plane.

The results shown in Figure 2 can be analyzed further using eq 2. Figure 3 shows illustrative solutions of the *full* (not asymptotic) BTC behavior for the dimensional Fickian diffusion case , eq 2, together with the asymptotic behavior for the dimensional anomalous diffusion case, eq 3, as shown also in Figure 2. The Fickian diffusion coefficients *D*_d_ selected here are listed in Table 1. These values of *D*_d_ are generally similar to those reported in other chalk diffusion studies;^28,32^ however, the *D*_d_ value for Silurian dolomite is an order of magnitude higher than “usual” estimates, further illustrating a deviation from expected Fickian diffusion.^45^

The plots in Figure 3 show that varying the value of *D*_d_ in the Fickian BTC (i) controls the duration until the first arrival of tracer—in this case, seen as first values of log_10_(1 – *C*/*C*_0_) < 0—as well as (ii) the rate of transition as the long-time tail falls to a slope of −0.5. Increasing *D*_d_ decreases the duration to first arrival and sharpens the rate of transition to the slope of −0.5. Clearly, though, the actual diffusion is anomalous, and solutions of the Fickian diffusion equation diverge significantly from experimental data at longer times. Moreover, from Figure 3, the measurements indicate that each rock core sample exhibited unique early arrival times, a (relatively short) duration over which diffusion could be interpreted as Fickian, and a unique transition toward anomalous diffusion. For both desert pink samples [plots (a,c)], the Fickian diffusion solution matches the initial diffusion behavior over ∼10 days. For Edwards yellow, the solution fits the initial diffusion for ∼18 days [plot (d)] and only ∼2 days [plot (b)], while for SL-PL, the solution fits the initial Silurian dolomite data for ∼4 days [plot (e)]. Finally, note that these plots also suggest a time frame for the experiment durations required to differentiate between Fickian and anomalous diffusion, wherein the Fickian solution deviates from the measurements. These results also confirm the need for experimental design that avoids blurring of the long-time, low concentration tailing.

### Insight and Implications

To provide physical/conceptual insight into why diffusion in porous rocks and sediments is—or can be—anomalous, we first refer to percolation theory and random walk considerations. Consider chemical species (“particle”) diffusion on an orthogonal (2D or 3D) lattice. Particle migration away from a source, on a fully connected lattice, for example, is at longer times generally much faster than on a poorly connected lattice near the percolation threshold (wherein the entire lattice is “just” connected across the domain). This occurs because diffusing particles become entrapped in dangling clusters and thus reduce the average migration away from the source.^46^ Extensive numerical simulations confirm that for a full lattice, the mean squared displacement traveled by diffusing particles scales linearly with time, ⟨*x*^2^(*t*)⟩ ∼ *t*, while on sparser networks, the scaling is a power law, ⟨*x*^2^(*t*)⟩ ∼ *t*^β^, with β becoming increasingly smaller (<1) as the lattice nears the percolation threshold.^46^ Similarly, numerical simulations of anomalous diffusion, in a domain with tracer movement defined via a generalization of Brownian motion, clearly show^47^ that as β (<1) becomes smaller in the context of *t*^β^ scaling, the diffusion pattern may become increasingly compact in space.

Anomalous diffusion can lead to significantly longer late-time arrivals relative to Fickian diffusion. This can have profound impacts on hydrogeological-geochemical studies, for example, in safety assessments that examine the slow but steady diffusive leaching of a contaminant away from the source and into the geosphere. These assessments are critical for groundwater contamination studies,^23,24,28,32,48,49^ for consideration of subsurface disposal of radioactive and toxic wastes, and anthropogenic CO_2_,^50−52^ and for the analysis and monitoring of chemical species mobility in river, lake, and marine sediments.^53−56^

In these contexts, a key, quantitative insight can be derived from consideration of Figure 3. Comparing values of *C*/*C*_0_ between (asymptotic) anomalous and (full) Fickian solutions, at a time of log_10_1.5 ∼ 32 days, we find that the estimates of *C*/*C*_0_ (converting from log_10_[1 – *C*/*C*_0_] on the plots), translate as follows, for the anomalous vs Fickian solutions, respectively: *C*/*C*_0_ ∼ 0.37 vs ∼0.57, *C*/*C*_0_ ∼ 0.47 vs ∼0.75, *C*/*C*_0_ ∼ 0.27 vs ∼0.53, *C*/*C*_0_ ∼ 0.55 vs ∼0.67, and *C*/*C*_0_ ∼ 0.30 vs ∼0.75, for Figures 3a–e, respectively. Clearly, at even longer times, the deviations between anomalous and Fickian diffusion behavior become ever greater, suggesting potential orders of magnitude differences in actual and calculated arrival times. Slower chemical migration and arrival at a monitoring point would suggest, for example, that efforts to remediate a contaminated aquifer may require times that are orders of magnitude larger than those estimated under the assumption that diffusive processes are Fickian.

### Emerging Directions for Future Research

Notwithstanding evidence of anomalous diffusion in a variety of other types of porous media (e.g., biological cells and membranes and dense liquids and gels^7−11,13−22^), there have not been, to date, specific measurements and analyses that search for it in similarly heterogeneous, disordered media such as soils, sediments, and rocks. Thus, the aim of the present work was to develop experiments to measure the diffusion of a conservative chemical species in a set of carbonate rock samples, with boundary conditions representing natural settings. The experiments demonstrate that diffusion in these rocks is anomalous, with long-time migration behavior distinctly different from that expected for classical Fickian diffusion. The anomalous behavior is quantified within a CTRW framework that accounts for broad distributions of diffusion times. The ability to quantify such behavior opens an important path to analyzing anomalous diffusion in rocks, soils, and sediments.

Noting that anomalous (non-Fickian) diffusion is likely the “normal” behavior in most real environmental settings in geological formations and marine, lake, river, and sediments, we conclude that reassessment of estimates of water chemistry evolution, predicated on the occurrence of classical Fickian diffusion, should be revisited.

## Data Availability

Ancillary measurements and equations used in this publication are provided in the manuscript.

## Supporting Information Available

Data from experiments (PDF)
