# Supplementary material for: Diffusion in Porous Rock Is Anomalous
Source: Environ Sci Technol. 2024 May 13;58(20):8946–54. doi: 10.1021/acs.est.4c01386 (PMC11112742; doi:10.1021/acs.est.4c01386)
Supplement: Supplementary file 1 — es4c01386_si_001.pdf [file es4c01386_si_001.pdf]

Supporting Information

Diffusion in Porous Rock is Anomalous

Ashish Rajyaguru,[*,†] Ralf Metzler,[‡] Ishai Dror,[†] Daniel Grolimund,[¶] and Brian Berkowitz[*,†]

[†]Department of Earth and Planetary Sciences, Weizmann Institute of Science, Rehovot 7610001, Israel
[‡]Institute for Physics and Astronomy, University of Potsdam, 14476 Potsdam, Germany ; and Asia Pacific Centre for Theoretical Physics, Pohang 37673, Republic of Korea
[¶]Paul Scherrer Institut, 5232 Villigen, Switzerland

E-mail: ashish.rajyaguru@psi.ch; brian.berkowitz@weizmann.ac.il

Summary: 11 pages, 5 tables.

Measurements shown in Figures 1a and 2a: Desert Pink, perpendicular to the bedding plane

$\log_{10}$(time), days	$\log_{10}(1-C/C_0)$
-0.3391	-0.0276
-0.2660	-0.0257
-0.2041	-0.0202
-0.1500	-0.0115
-0.1051	-0.0109
-0.0615	-0.0122
0.0026	-0.0098
0.0374	0.0001
0.0693	0.0038
0.1065	-0.0006
0.1339	0.0045
0.1596	0.0075
0.1821	0.0084
0.2071	0.0043
0.2274	0.0007
0.2874	-0.0039
0.3400	-0.0138
0.3881	-0.0295
0.4681	-0.0327
0.5043	-0.0409
0.5371	-0.0416
0.5668	-0.0429
0.5953	-0.0428
0.6220	-0.0562
0.6472	-0.0630
0.6710	-0.0762
0.7150	-0.0488
0.7550	-0.0509
0.7744	-0.0874
0.7973	-0.1106
0.8198	-0.0872
0.8419	-0.0801
0.8616	-0.1117
0.8810	-0.0856
0.9008	-0.1019
0.9176	-0.1042
0.9347	-0.1070
0.9539	-0.0780
1.0192	-0.0885
1.0414	-0.1351
1.0607	-0.0670
1.0811	-0.1306
1.0984	-0.1415
1.1171	-0.0820
1.1490	-0.1030
1.1800	-0.1043

1.2081	-0.0851
1.2342	-0.1847
1.2583	-0.2387
1.2826	-0.1600
1.3053	-0.1588
1.3252	-0.1660
1.3465	-0.2118
1.3835	-0.1647
1.4020	-0.1637
1.4184	-0.0970
1.4344	-0.1867
1.4509	-0.2372
1.4658	-0.1735
1.4807	-0.2510
1.4942	-0.1779
1.5079	-0.2326
1.5213	-0.1580
1.5339	-0.1794
1.5465	-0.1942
1.5589	-0.2206
1.5695	-0.2461
1.5817	-0.2044
1.5935	-0.2248
1.6044	-0.2310
1.6149	-0.2241
1.6253	-0.1989
1.6355	-0.2203
1.6454	-0.2378
1.6551	-0.3042
1.6645	-0.3123
1.6740	-0.1648
1.6830	-0.2328
1.6921	-0.2668
1.7006	-0.1836
1.7092	-0.2211
1.7177	-0.2267
1.7262	-0.2292
1.7346	-0.2152
1.7431	-0.1712
1.7511	-0.2353
1.7662	-0.2322
1.7741	-0.2390
1.7810	-0.1878

Measurements shown in Figures 1b and 2b: Edwards Yellow, perpendicular to the bedding plane

log_{10}(time), days	$log_{10}(1-C/C_0)$
-0.5051	-0.0278
-0.1899	-0.0387
0.0060	-0.0486
0.1213	-0.0551
0.2173	-0.0575
0.3078	-0.0601
0.3680	-0.0695
0.4299	-0.0677
0.4816	-0.0708
0.5287	-0.0776
0.5824	-0.0925
0.6241	-0.1069
0.6615	-0.1084
0.7404	-0.0836
0.7831	-0.0969
0.8490	-0.1134
0.8515	-0.1319
0.8905	-0.1477
0.9098	-0.1572
0.9326	-0.1665
0.9582	-0.1792
0.9806	-0.1891
1.0042	-0.1943
1.0253	-0.1913
1.0490	-0.1917
1.0854	-0.1956
1.1188	-0.2131
1.1512	-0.2150
1.1805	-0.2059
1.2084	-0.2155
1.2342	-0.2252
1.2570	-0.2426
1.2817	-0.2257
1.3049	-0.2299
1.3242	-0.2541
1.3456	-0.2198
1.3647	-0.2131
1.3831	-0.2390
1.4007	-0.2326
1.4176	-0.2192
1.4338	-0.2051
1.4497	-0.2295
1.4647	-0.2308
1.4796	-0.2542

1.4934	-0.2619
1.5072	-0.2508
1.5340	-0.2240
1.5471	-0.2015
1.5600	-0.2035
1.5721	-0.1988
1.5840	-0.2085
1.5949	-0.2291
1.6065	-0.2372
1.6166	-0.2262
1.6275	-0.2379
1.6375	-0.2509
1.6474	-0.2660
1.6569	-0.2843
1.6663	-0.3115
1.6939	-0.3145
1.7560	-0.2954
1.7718	-0.2923
1.7866	-0.2996
1.8025	-0.2971
1.8143	-0.3135
1.8337	-0.3296
1.8481	-0.3179

Measurements shown in Figures 1c and 2c: Desert Pink, parallel to the bedding plane

$\log_{10}$(time), days	$\log_{10}(1-C/C_0)$
-0.3227	-0.0233
-0.1397	-0.0214
0.0119	-0.0099
0.1005	-0.0216
0.3151	-0.0222
0.3647	-0.0192
0.4021	-0.0333
0.4360	-0.0218
0.4924	-0.0147
0.5247	-0.0218
0.5655	-0.0282
0.6118	-0.0333
0.6417	-0.0374
0.6732	-0.0408
0.7138	-0.0471
0.7404	-0.0506
0.7669	-0.0529
0.7908	-0.0598
0.8148	-0.0594
0.8368	-0.0622
0.8541	-0.0627
0.8757	-0.0584
0.8964	-0.0630
0.9132	-0.0654
0.9296	-0.0634
0.9492	-0.0664
0.9637	-0.0671
0.9780	-0.0800
0.9967	-0.0766
1.0104	-0.0783
1.0209	-0.0779
1.0407	-0.0868
1.0522	-0.0934
1.0608	-0.0895
1.0759	-0.0857
1.0878	-0.0861
1.0968	-0.0837
1.1149	-0.0895
1.1312	-0.0858
1.1159	-0.0903
1.1627	-0.0909
1.1805	-0.0955
1.1939	-0.0940
1.2092	-0.0890
1.2209	-0.0933
1.2337	-0.0980

1.2209	-0.1049
1.2582	-0.1045
1.2825	-0.1040
1.3092	-0.1075
1.3430	-0.1153
1.3831	-0.1223
1.4183	-0.1299
1.4544	-0.1370
1.5210	-0.1423
1.5502	-0.1426
1.5911	-0.1550
1.6250	-0.1487
1.6641	-0.1621
1.6990	-0.1685
1.7241	-0.1764
1.7574	-0.1847
1.8066	-0.1681

Measurements shown in Figures 1d and 2d: Edwards Yellow, parallel to the bedding plane

$\log_{10}$(time), days	$\log_{10}(1\text{-}C/C_0)$
-0.1397	0.0041
0.0119	0.0160
0.1005	0.0082
0.1771	-0.0146
0.2378	-0.0378
0.3151	-0.0382
0.3647	-0.0605
0.4021	-0.0864
0.4360	-0.0779
0.4924	-0.0959
0.5247	-0.0868
0.5655	-0.1125
0.6118	-0.1405
0.6417	-0.1536
0.6732	-0.1337
0.7138	-0.1999
0.7404	-0.1986
0.7669	-0.1797
0.7908	-0.2092
0.8148	-0.1785
0.9132	-0.2392
0.9296	-0.2339
0.9492	-0.2292
0.9637	-0.2434
0.9780	-0.2004
0.9967	-0.2387
1.0104	-0.2671
1.0209	-0.2720
1.0407	-0.2016
1.0522	-0.2892
1.0608	-0.2972
1.0759	-0.2642
1.0878	-0.3252
1.0968	-0.2730
1.1149	-0.2305
1.1312	-0.2478
1.1159	-0.2380
1.1627	-0.2574
1.1805	-0.2309
1.1939	-0.2532
1.2092	-0.2975
1.2209	-0.3015
1.2337	-0.3459
1.2209	-0.3267
1.2582	-0.3081
1.2825	-0.2956

1.3092	-0.3238
1.3430	-0.3509
1.3831	-0.3380
1.4183	-0.3314
1.4544	-0.3009
1.4803	-0.3504
1.5210	-0.3415
1.5502	-0.3940
1.5911	-0.4028
1.6250	-0.3413
1.6641	-0.4082
1.6990	-0.3738
1.7241	-0.3704
1.7574	-0.3911
1.7782	-0.3809
1.8066	-0.3621
1.8265	-0.3788

Measurements shown in Figures 1e and 2e: Silurian Dolomite, parallel to the bedding plane

$\log_{10}$(time), days	$\log_{10}(1\text{-}C/C_0)$
-0.3227	-0.0149
-0.1397	-0.0422
0.0119	-0.0431
0.1005	-0.0603
0.1771	-0.0899
0.2378	-0.0984
0.3151	-0.1042
0.3647	-0.1251
0.4021	-0.1270
0.4360	-0.1337
0.4924	-0.1444
0.5247	-0.1559
0.5655	-0.1495
0.6118	-0.1649
0.6417	-0.1234
0.6732	-0.1658
0.7138	-0.1506
0.7404	-0.1263
0.7669	-0.1233
0.7908	-0.1662
0.8148	-0.1464
0.8368	-0.1118
0.8541	-0.1387
0.8757	-0.1682
0.8964	-0.1027
0.9132	-0.1123
0.9296	-0.1391
0.9492	-0.1762
0.9637	-0.1567
0.9780	-0.1257
0.9967	-0.1166
1.0104	-0.1486
1.0209	-0.1272
1.0407	-0.1301
1.0522	-0.1475
1.0608	-0.1426
1.0759	-0.1333
1.0878	-0.1483
1.0968	-0.1509
1.1149	-0.1527
1.1312	-0.1496
1.1159	-0.1441
1.1627	-0.1680
1.1805	-0.1370
1.1939	-0.1563
1.2092	-0.1598

1.2209	-0.1530
1.2337	-0.1700
1.2209	-0.1592
1.2582	-0.1594
1.2825	-0.1517
1.3092	-0.1463
1.3430	-0.1645
1.3831	-0.1600
1.4183	-0.1548
1.4544	-0.1658
1.4803	-0.1424
1.5210	-0.1431
1.5502	-0.1476
1.5911	-0.1607
1.6250	-0.1526
1.6641	-0.1457
1.6990	-0.1636
1.7241	-0.1769
1.7574	-0.1487
1.7782	-0.1599
1.8066	-0.1700
